# The Utilisation of *Fucus vesiculosus* Algae Extracts in the Production of Microgreens *Hordeum vulgare* L. with an Increased Content of Selected Bioactive Compounds

**DOI:** 10.3390/plants13202871

**Published:** 2024-10-14

**Authors:** Barbara Drygaś, Tomasz Piechowiak, Joanna Kreczko, Natalia Matłok, Bogdan Saletnik, Maciej Balawejder

**Affiliations:** 1 Department of Bioenergetics, Food Analysis and Microbiology, University of Rzeszow, 35-601 Rzeszow, Poland; 2 Department of Chemistry and Food Toxicology, University of Rzeszow, 35-601 Rzeszow, Poland; tpiechowiak@ur.edu.pl (T.P.); mbalawejder@ur.edu.pl (M.B.); 3 Utrica Technologies Sp. z o.o., ul. Stanisława Lema 4A/1, 80-126 Gdańsk, Poland; info@urti-tech.pl; 4 Department of Food and Agriculture Production Engineering, University of Rzeszow, 35-601 Rzeszow, Poland; nmatlok@ur.edu.pl

**Keywords:** biostimulants, bioactive compounds, microgreens, algae extracts, antioxidant activity

## Abstract

Algae extracts may be a promising alternative to harmful chemicals and pesticides used commercially in the cultivation of plants with higher nutritional and health-promoting values. The cultivation of barley microgreens (*Hordeum vulgare* L.) was facilitated by the use of aqueous extracts from *Fucus vesiculosus* algae, which served as a biostimulant. Seeds for experiments were produced in accordance with EU standards, certified as organic and used to grow plants in a controlled pot experiment. A qualitative analysis of the extract, which was used to irrigate the plants, was also performed in this study, as well as stimulating properties by activating the system protecting against oxidative stress. Total phenolic content (TPC), total flavonoid content (TFV) and enzymes involved in their formation such as phenylalanine ammonia lyase (PAL) and polyphenol oxidase (PPO), as well as enzymes involved in the removal of reactive oxygen species such as catalase (CAT) and superoxide dismutase (SOD), were determined in the obtained microgreen samples. Antioxidant activity against DPPH (2,2-diphenyl-1-picrylhydrazyl) was also evaluated. A noticeable increase in SOD content and antioxidant activity against DPPH was observed in barley microgreen samples after extract treatment. These results suggest that the use of extracts of this beneficial alga can enhance the antioxidant activity of the barley microgreens.

## 1. Introduction

Growing demand for food and the fear of excessive chemical inputs are driving agriculture to seek sustainable solutions for increasing crop yields in the face of global change [1,2,3]. Research into brown algae, which produce beneficial compounds, is exploring their effects on plant growth and stress resistance [1,4,5]. New commercial biostimulant products are emerging that stimulate plants and enhance soils to alleviate plant stressors such as drought and frost [1]. While the efficacy of many has been demonstrated, the exact relationship between extract composition and its effects still remains unclear, but molecular synergy is thought to play a key role [1,6].

Brown algae (*Phaeophyceae*) are gaining increasing attention as a promising source of biostimulants for sustainable agriculture [1,7].

They contain a wide range of bioactive compounds such as osmoregulatory, antioxidant, pro-bacterial and plant growth-promoting substances [1].

It is hypothesised that the compounds present in brown macroalgae extracts act as signalling molecules, affecting mechanisms for specific metabolic and hormonal pathways, modulating gene expression and inducing metabolic changes in the plant [1,8]. In response to stress, these pathways can be amplified to enhance the plant’s capacity for adaptation and survival, or to delay the effects of stress. Moreover, some of these compounds have been demonstrated to possess antioxidant properties [1,9]. Given its complex nature, it is suspected that minor alterations in the composition of the extract can result in disparate molecular and cellular outcomes. Moreover, a single extract can simultaneously activate and/or inhibit multiple metabolic or hormonal pathways. Therefore, it is not possible to make any generalisations regarding the mode of action and subsequent benefits based solely on the biological origin of the extracts [1,10].

*Fucus vesiculosus* L. (*Fucaceae*), commonly referred to as bladderwrack, is a marine organism that contains a variety of biologically active compounds. These include specific phenolic compounds called phlorotannins, the carotenoid fucoxanthin, minerals (including iodine and calcium) and bioactive polysaccharides, namely fucoidans, laminarans and alginates. These compounds have been shown to possess a range of important biological properties [11,12,13,14]. Furthermore, it has been established that the biochemical composition of *F. vesiculosus* exhibits variability contingent on the geographical location, developmental stage or stress factors for the organism in question [15,16]. The algae from the Barents Sea had the highest levels of xylose and fucose during the fertile phase. There was a positive correlation between the build-up of monosaccharides, phlorotannins and flavonoids and water salinity and a negative correlation between radical scavenging activity and the amount of structural fucoidan monosaccharides. The elemental content of *F. vesiculosus* from different Arctic seas was also studied. It was found that this alga does not express the tendency to accumulate toxic doses of metallic pollutants. The researchers concluded that *F. vesiculosus* can be safely used in food and medicine [15]. Other species from the *Fucaceae* family, such as *Ascophyllum nodosum*, have been shown to stimulate plant growth and development when applied to seeds or plants [17,18,19]. With this in mind, it seemed interesting to test whether *F. vesiculosus* extracts could potentially increase the nutritional value of microgreens. Sequential extraction approaches can selectively recover specific chemical compounds from bladderwrack. Using water in the first step yields water-soluble compounds, like phlorotannins, laminarans and mannuron-rich alginates, while ethanol extraction maximises fucoxanthin recovery. This allows the composition of the extract to be tailored for specific applications [14]. The preliminary extraction of compounds from food sources using water at room temperature is an environmentally friendly and cost-effective process. It yields food-grade extracts with high concentrations of specific compounds that could be utilised in functional foods [14].

The extraction process of algae depends on the desired end product. Thermochemical conversion using high temperatures and solvents is still used industrially, but has a negative impact on the environment and product functionality [20]. Advanced green extraction techniques isolating bioactive compounds from marine macroalgae are faster, more sustainable and efficient. They reduce the consumption of organic solvents, which allows for avoiding their toxic effects. In addition, cell wall disruption techniques, including mechanical technologies (MAE, UAE and UMAE), can significantly improve extraction efficiency and reduce extraction time [21].

Ultrasound-assisted extraction (UAE) is a technique, regarded as a “clean technology”, that has garnered attention in recent years due to its notable advantages over conventional techniques. These include the use of minimal solvent volumes, brief extraction times, reduced instrumental requirements and a low economic and environmental impact [22]. The use of ultrasound has been shown to enhance the extraction of high molecular weight phenolic compounds, as evidenced by the analysis of *A. nodosum* extracts. Consequently, ultrasound-assisted extraction (UAE) can be employed to improve the recovery of bioactive compounds from seaweeds [23].

While seaweed extracts may enhance plant growth, further research is needed to explore the potential of *F. vesiculosus* extracts in the production of nutrient-rich barley microgreens. The specific use of bladderwrack extracts for the production of nutrient-rich barley (*Hordeum vulgare* L.) microgreens has not been reported in the literature. There are not many publications directly describing the use of *F. vesiculosus* extracts in microgreen cultivation.

It has been demonstrated that *A. nodosum*, a different species of brown seaweed, is capable of influencing the levels of certain bioactive compounds in plants [17,18,19].

The objective of this study was to evaluate the impact of *Fucus vesiculosus* algae extracts on the concentration of specific bioactive compounds in barley (*Hordeum vulgare* L.) microgreens. The study aimed to assess whether irrigation with bladderwrack extract can increase the content of phenolic compounds, flavonoids and antioxidant activity, as well as the activity of enzymes such as catalase (CAT), polyphenol oxidase (PPO), phenylalanine ammonia lyase (PAL) and superoxide dismutase (SOD) in barley microgreens.

## 2. Results

**Figure 1:**
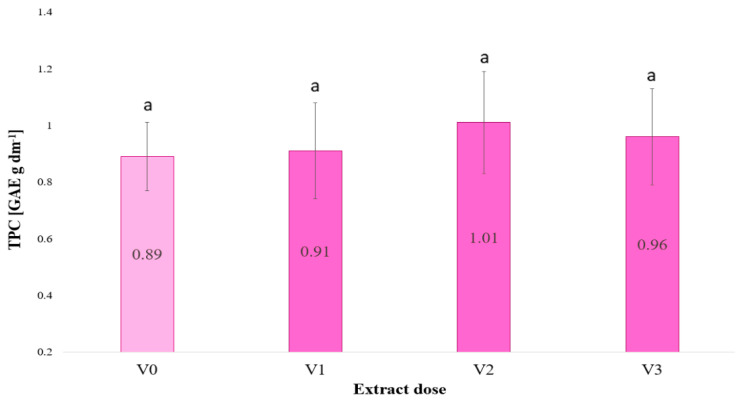
Total phenolic compounds (TPC) in barley microgreens as a function of the dose of *Fucus vesiculosus* extract used to irrigate the plants (*n* = 20); significant differences between results are indicated by lower case letters; significance level was defined as *p* < 0.05.

**Figure 2:**
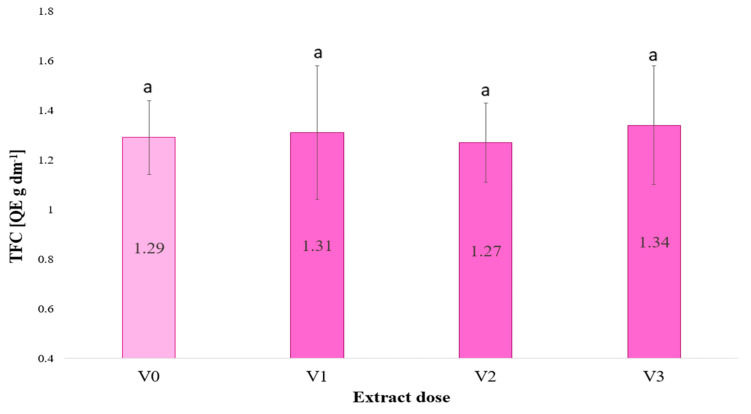
Total flavonoids content (TFC) in young *Hordeum vulgare* L. microgreens as a function of the dose of *Fucus vesiculosus* extract used to irrigate the plants (*n* = 20); significant differences between results are indicated by lower case letters; significance level was defined as *p* < 0.05.

**Figure 3:**
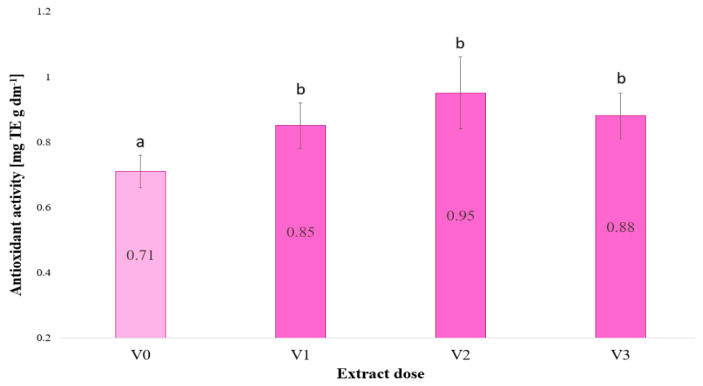
Antioxidant activity against DPPH in young barley plants as a function of the dose of algae extract used to irrigate the plants (*n* = 20); significant differences between results are indicated by lower case letters; significance level was defined as *p* < 0.05.

**Figure 4:**
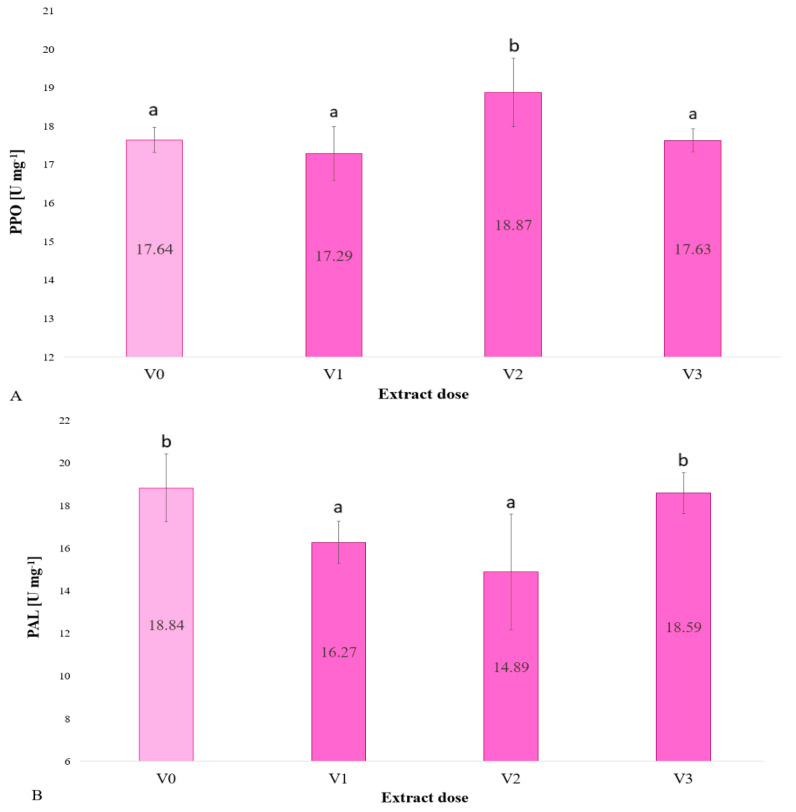
Polyphenol oxidase (PPO) (**A**) and phenylalanine ammonia lyase (PAL) (**B**) in barley microgreen plants as a function of the dose of extract used to irrigate the plants (*n* = 20); differences between results are indicated by lower case letters; significance level was defined as *p* < 0.05.

**Figure 5:**
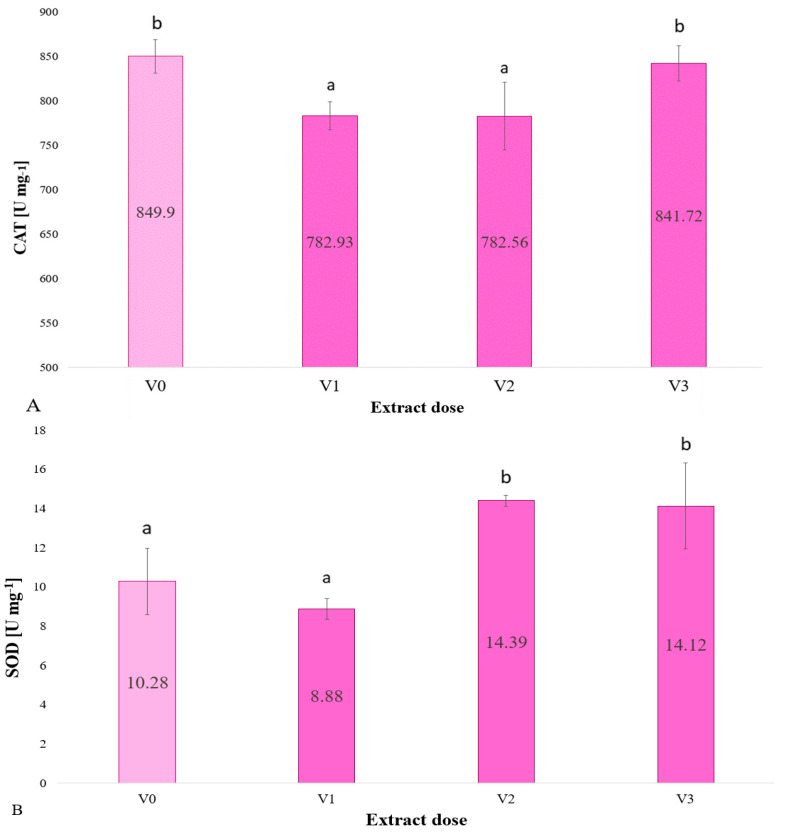
Catalase (CAT) (**A**) and superoxide dismutase (SOD) (**B**) activities in barley microgreens in relation to the dose of extract used to water the plants (*n* = 20); differences between results are indicated by lower case letters; significance level was defined as *p* < 0.05.

In this study, the composition of the aqueous extract of *F. vesiculosus* was evaluated (Table 1)and selected enzymes and antioxidant activity were assessed (Figure 1, Figure 2, Figure 3, Figure 4 and Figure 5).

### 2.1. Assay of Microgreens

#### 2.1.1. Total Phenolic Compounds, Flavonoids and Antioxidant Activity against DPPH

In the presented study, the total phenolic compound content of the experimental objects was assessed. The results demonstrated that irrigation with *F. vesiculosus* extract had no significant impact on the total phenolic compound (TPC) content in barley microgreens (see Figure 1).

Figure 1 shows the total phenolic content in young barley plants as a function of the dose of *F. vesiculosus* extract used for irrigation. No significant differences were observed in the ability of the doses to increase phenolic content.

The experiment conducted shows that the active compounds in the *F. vesiculosus* extract are not sufficiently effective in stimulating the production of phenolic compounds in barley.

The flavonoid content (Figure 2) of young barley leaves was found to remain unchanged when irrigating with the extract. The possible reasons for this are analysed in the “Discussion” Section.

Figure 3 illustrates the antioxidant activity of barley microgreens, expressed in Trolox equivalents per gram of dry weight. The lowest value of antioxidant activity against DPPH was observed in the control (V0) treatment, indicating that the absence of irrigation with the extract resulted in the lowest antioxidant activity. The antioxidant activity of all plants irrigated with the extracts (at concentrations of 2.5, 5 and 10%) was found to be higher than that of the control, indicating that the *F. vesiculosus* extract enhances antioxidant activity. An increase in the dose of the extract resulted in a corresponding rise in antioxidant activity, reaching a maximum at 5% (0.95). At 10%, the antioxidant activity exhibited a slight decline in comparison to V2, yet remained higher than that of the control.

#### 2.1.2. Phenylalanine Ammonia Lyase and Polyphenol Oxidase Activities

Figure 4A shows the activity of the polyphenol oxidase (PPO) enzyme in units per milligram. The PPO activity in the control group was 7.64, which serves as a reference point for other doses. The PPO activity of plants treated with the V1 dose (2.5% extract concentration) was observed to be slightly lower than that of the control, though the difference was not found to be statistically significant. The highest PPO activity was observed in plants treated with a 5% extract, and this difference was found to be statistically significant in comparison to the control and another doses. For the 10% extract concentration, the PPO activity is comparable to that of the control, with no statistically significant discrepancy.

Figure 4B shows how phenylalanine ammonia lyase content in barley microgreens irrigated with brown algae extract changes. The control and the V3 dose (10%) had the highest PAL values. Irrigating with the extract at 2.5 and 5% reduced PAL activity. The PAL value at 10% was similar to the control, suggesting that this dose does not affect PAL activity.

#### 2.1.3. Catalase and Superoxide Dismutase Activities

In the presented study, an increase in SOD concentration was observed in barley microleaves under the influence of the application of *F. vesiculosus* extract at a concentration of 5 and 10% (see Figure 5B).

Figure 5A shows the catalase activity (in units per milligram of biomass, U) for different doses of the extract, labelled V1, V2 and V3, where V0 is the control. The highest catalase activity was observed at dose 0 (in the control, −849.90 U mg). The lowest catalase activity was observed at dose 5% (782.93 U mg). Doses V2 and V3 (5 and 10%) have similar values of catalase activity, 785.56 and 841.72 U mg B1, respectively. Doses 2.5% and 5% are not significantly different from each other, but are significantly different from the control and 10%. The control and 10% doses are also not significantly different from each other, but are significantly different from 2.5% and 5%. In summary, the 2.5% and 5% doses resulted in a decrease in catalase activity compared to the control, while the 10% dose did not significantly change catalase activity compared to the control.

The highest superoxide dismutase (SOD) levels were observed for the V2 and V3 doses (5 and 10% extract), indicating that these doses of the extract are the most efficacious in increasing SOD levels. The control plants and those administered the lowest dose (V1, 2.5%) exhibited reduced SOD levels. The effects of the doses of the extracts on the plants may vary depending on their composition and concentration. The 2.5% extract concentration appears to exert the least effect on SOD levels, indicating that it may be less efficacious in stimulating enzyme activity. The data indicate that plants administered the 5 and 10% doses exhibited elevated SOD levels.

## 3. Discussion

Brown algae warrant consideration as a novel and promising source of biologically active compounds with diverse potential applications in the food, pharmaceutical and cosmetic industries [24,25,26,27]. These species remain under investigation and screening for biomolecules, particularly the phenolic group of phlorotannins, which are not found in terrestrial sources. Consequently, the isolation, identification and pharmacological characterisation of these compounds represent emerging scientific interests, necessitating the adoption of novel and sustainable approaches [24].

Phenolic compounds represent the group of metabolites with the greatest structural variation and the highest content in seaweeds [28]. The most extensively researched seaweed polyphenol class is the phlorotannins, which are synthesised by brown seaweeds. However, there are other polyphenolic compounds, including bromophenols, flavonoids, phenolic terpenoids and mycosporine-like amino acids. The compounds that have already been found and characterised demonstrate a wide range of bioactivities and potential future applications in various industrial sectors [28]. Seaweed collected after being washed up on beaches is an untapped natural resource with potential uses in cosmetics, pharmacy, fertilisers and sustainable agriculture. Bikovens et al. [29] showed that *F. vesiculosus* could be used to make nitrogen-containing fertilisers and biologically active compounds. The ethyl acetate extract was rich in phenolic compounds and had high antioxidant activity. The ethanol extract expressed high amounts of phlorotannins, as shown by the LC-MS/MS analysis.

In the presented study, the results demonstrated that irrigation with *F. vesiculosus* extract had no significant impact on the TPC in barley microgreens. In a study conducted by Babazadeh et al. [30], the impact of a brown alga from the *Phaeophyceae* class on cold tolerance in barley plants was investigated. In this study, the impact of an aqueous extract of *Sargassum angustifolium* (*Sargassaceae, Phaeophyceae*) at a concentration of 0.05% (*w*/*v*) on *Hordeum vulgare* L. plants cultivated hydroponically under control conditions or under cold stress for a period of two weeks was investigated. In the aforementioned experiment, the utilisation of *S. angustifolium* extract resulted in an increase in plant biomass under control conditions. Conversely, algae extracts did not affect the biomass under cold stress. The extract demonstrated a positive impact on photosynthetic parameters, including leaf pigments and chlorophyll fluorescence, as well as biochemical stress indicators such as phenolic compounds, peroxidase activity and ammonia-phenylalanine lyase activity [30].

The results of the experiment conducted demonstrate that the active compounds present in the *F. vesiculosus* extract are not sufficiently efficacious in stimulating the production of phenolic compounds in *Hordeum vulgare* L. Barley microgreens may not respond to the *F. vesiculosus* extract in the same way as other plants because of metabolic and environmental differences as well as differences in signalling pathways.

Although different doses have been tested, it is possible that the optimum dose has not yet been identified. As mentioned above, molecular synergy may play a key role. The lack of significant differences may be due to a lack of synergy between the extract components and the plant metabolites.

The experiment showed that the flavonoids in barley microgreens did not change as a result of treatment with the extract. The authors, in an attempt to explain this phenomenon, have taken into consideration various alternatives, which are yet to be tested.

The barley microgreens may have special ways of defending themselves that do not respond to the compounds in bladderwrack aqueous extracts. *F. vesiculosus* extracts may lack the key components to affect flavonoid biosynthesis in barley. It is also possible that the compounds in the extracts do not interact with the enzymes responsible for flavonoid production in barley. Environmental conditions may also affect flavonoid levels more than the seaweed extracts.

Plants have a developed antioxidant defence system comprising a variety of enzymes that are vital for overcoming various types of stress. The main plant enzymes are superoxide dismutase (SOD), catalase (CAT), peroxidase (POX), glutathione peroxidase (GPX), glutathione reductase (GR), glutathione S-transferases (GST), ascorbate peroxidase (APX), monodehydroascorbate reductase (MDHAR) and dehydroascorbate reductase (DHAR). These function as part of the antioxidant defence system. Together, these enzymes form a complex set of mechanisms that effectively minimise, buffer and remove reactive oxygen species (ROS) [31].

The DPPH method is a widely employed approach for assessing antioxidant activity in plants [32,33]. The experiment conducted showed that the antioxidant activity against DPPH in young barley plants increased as a result of their irrigation with a solution of *F. vesiculosus* algae.

In the presented study, the control (V0) had the lowest antioxidant activity against DPPH, showing that not irrigating with the extract resulted in the lowest antioxidant activity. The plants irrigated with the *F. vesiculosus* extract had higher antioxidant activity than the control. More extract means more antioxidant activity. The maximum was 5% (0.95). At 10%, antioxidant activity decreased slightly compared to V2, but remained higher than in the control. The decrease in antioxidant activity at the V3 dose compared to V2 may be due to a number of factors. At higher extract doses, further amounts of extract may not increase antioxidant activity. Furthermore, increased extract concentrations may result in unfavourable chemical interactions that potentially reduce antioxidant efficacy.

Additionally, exceedingly high doses of the extract may induce toxic effects that could impair cellular function and diminish overall antioxidant activity. Furthermore, it is suspected that the application of higher concentrations of the extract may result in alterations to the bioavailability of the active ingredients, which could potentially impact their efficacy.

In the experiment conducted by Drygaś et al. [34] on an arugula under similar conditions, the application of aqueous extracts of the brown alga *A. nodosum* also resulted in an increase in antioxidant activity (DPPH).

Phenylalanine Ammonia Lyase and Polyphenol Oxidase Activities. Polyphenol oxidase (PPO) is an oxidoreductase enzyme. In damaged plant tissues, it catalyses the enzymatic browning process by oxidising o-diphenols to highly reactive o-quinones, which then polymerise to produce heterogeneous dark polymer melanin [35].

In a study conducted by Babazadeh et al. [30], the absence of algal extract had no effect on PAL activity in the absence of chilling stress; however, in plants treated with *Sargassum angustifolium*, PAL activity was significantly increased. In contrast, the activity of PPO was found to significantly decrease under conditions of chilling stress, with a further reduction observed upon the application of algal extract to plants subjected to chilling stress. Similarly, the concentration of phenolics was not influenced by chilling stress; however, the algal extract markedly elevated this parameter in chilling-stressed plants [30].

The present study observed a slight reduction in PPO activity in plants treated with a 2.5% extract concentration, though this difference was not found to be statistically significant. The highest PPO activity was observed in plants treated with a 5% extract, and this difference was found to be statistically significant in comparison to the control and other doses. For the 10% extract concentration, the PPO activity was comparable to that of the control, with no statistically significant discrepancy.

In the experiment by Drygaś et al. [34] with the brown alga *A. nodosum*, PPO activity also increased in arugula microgreens, in particular at an extract concentration of 5% as well. The plants were grown under similar conditions and the extracts were prepared using the same protocol.

The experiment showed how the content of phenylalanine ammonia lyase changed in barley microgreens irrigated with brown algae extract. The control and 10% dose had the highest PAL values. The application of the extract at concentrations of 2.5 and 5% resulted in a reduction in PAL activity. The PAL value at 10% was observed to be comparable to that of the control, indicating that this dose does not exert an influence on PAL activity. The authors suspect that the intermediate doses may reduce enzyme activity because they are strong enough to elicit a biological response but not high enough to trigger defence mechanisms.

Catalase and Superoxide Dismutase Activities. SODs constitute a family of antioxidant enzymes that represent the primary line of defence against oxidative damage, and are present in every cell of all plant types [36,37,38]. Superoxide dismutase (SOD) turns superoxide anion (•O_2_^−^) into hydrogen peroxide (H_2_O_2_) and oxygen (O_2_). This reaction involves oxidation and reduction in the same reactant. Two compounds are formed: O_2_ (higher oxidation state) and H_2_O_2_ (lower oxidation state). Enzymes such as CAT work with SOD to prevent the formation of harmful ROS [31].

The presented study revealed an increase in SOD concentration in barley midleaves following the application of *F. vesiculosus* extract at concentrations of 5 and 10%.

The impact of seaweed extracts on SOD levels in young plants can be attributed to a number of factors. The bioactive compounds present in seaweed, including polysaccharides, amino acids, vitamins and phytohormones (auxins, cytokinins and gibberellins), can enhance plant resilience to environmental stress. In response to stress, plants can increase SOD production to neutralise reactive oxygen species. The application of seaweed extracts can also improve plant metabolism, thereby facilitating the better management of oxidative stress. Increased photosynthetic activity and improved nutrient uptake can also contribute to higher SOD levels. Seaweed stimulants can accelerate plant recovery after stress, which may include the increased production of antioxidant enzymes such as SOD.

The results of the experiment demonstrated that the control group exhibited the highest catalase activity. The lowest catalase activity was observed at a dose of 5%. The catalase activity values for doses V2 and V3 (5 and 10%, respectively) were found to be similar. The 2.5% and 5% doses exhibited no significant difference from one another; however, they were significantly different from the control and 10% doses. Furthermore, the control and 10% doses are not significantly different from each other, but are significantly different from the 2.5% and 5% doses. In conclusion, the 2.5% and 5% doses resulted in a reduction in catalase activity in comparison to the control, whereas the 10% dose did not exhibit a significant change in catalase activity in comparison to the control. There may be several reasons for this result. It is possible that at the highest concentration of extract (10%), the catalase enzyme has already reached its maximum activity and further increases in the dose of the extract do not lead to an increase in enzyme activity. Another reason for such a result could be enzyme inhibition. High concentrations of the extract may contain substances that act as catalase inhibitors, which may cause the enzyme activity to decrease to a level similar to that of the control. The highest concentration of extract applied to the plants may also have had a toxic effect on cells and enzymes, resulting in a decrease in catalase activity.

A study by Mansori et al. [39] demonstrated that extracts of *Ulva rigida* and *Fucus spiralis* algae applied to bean plants under drought conditions improved vegetative growth and increased stress tolerance. This extract reduced the impact of water deficit and enhanced the antioxidant potential of plants by activating the antioxidant enzyme systems SOD, CAT and APX and increasing the total phenolic content.

The highest SOD levels were observed for the 5 and 10% extracts, indicating that these doses of the extract are the most efficacious in increasing the levels of this enzyme. The control plants and those administered the lowest dose (2.5%) demonstrated a reduction in SOD levels. The impact of the doses of the extracts on the plants may be contingent upon their composition and concentration. The 2.5% extract concentration appears to exert the least effect on SOD levels, indicating that it may be less efficacious in stimulating enzyme activity. The administration of 5 and 10% doses of the *F. vesiculosus* aqueous extract to microgreens resulted in elevated SOD levels, indicating that higher concentrations of the extract are more efficacious in augmenting enzyme activity. Higher SOD levels may signify the enhanced protection of the plants from oxidative stress. These findings suggest that higher doses of the extract are more efficacious in stimulating plant defence mechanisms, which may culminate in superior resilience to environmental stresses.

In a study conducted by Babazadeh et al. [30], the application of an aqueous extract derived from the brown alga *Sargassum angustifolium* did not significantly affect the activity of SOD in young barley, when subjected to different temperature treatments. Similarly, the activity of catalase remained unaffected by any of the applied treatments [30]. The absence of a response from SOD and CAT activities to the algal extract does not preclude their involvement in maintaining ROS homeostasis in the extract-treated plants. It is plausible that, akin to the impact of cold stress, the algal extract may have failed to elicit a sustained augmentation in the enzyme [30].

## 4. Materials and Methods

### 4.1. Reagents and Laboratory Equipment

Biochemical and chemical reagents were obtained from Merck (Darmstadt, Germany), i.e., 2,2-diphenyl-1-picrylhydrazyl, gallic acid (97.5–102.5%), quercetin (analytical standard), trolox (97%), epinephrine, L-phenylalanine (≥99.0%), pyrocatechol, Triton X-100 and protease inhibitor cocktail (for general use), and Chempur (Piekary Śląskie, Poland), i.e., Folin–Ciocatleu reagent, sodium carbonate (analytical purity), aluminium chloride (analytical purity), sodium nitrite (analytical purity), sodium hydroxide (analytical purity), sodium chloride (analytical purity), hydrochloric acid (analytical purity), ammonium metavanadate (analytical purity), hydrogen peroxide (30%), sulphuric acid (analytical purity), ferric chloride (analytical purity), gelatin, sodium hydroxide (analytical purity), hydrochloric acid, Mayer’s reagent (potassium mercuric iodide solution), Wagner’s reagent (a solution of iodine in potassium iodide), Dragendorff’s reagent (potassium bismuth iodide solution), basic bismuth nitrate (analytical purity), tartaric acid (analytical purity), potassium iodide (analytical purity), glacial acetic acid (analytical purity), acetic anhydride (analytical purity), chloroform (analytical purity), Fehling’s A and Fehling’s B solutions, copper sulphate solution (analytical purity), nitric acid (analytical purity), ammonium hydroxide (analytical purity), Molisch’s reagent (α-naphthol 1% (*w*/*v*) in 90% ethanol) and an ultrasonic bath (CNCTech, Poland).

### 4.2. Preparation and Assays of Extracts

All extracts were prepared from commercially available raw materials (Dary Podlasia, Bielsk Podlaski, Poland) in the form of dried *F. vesiculosus* algae. The extracts were prepared using the UAE technique, following the same procedure as in the Drygaś et al. paper [34]. The initial extract was subsequently analysed and employed in the preparation of dilutions. The irrigation of the plants was conducted using preparations of the extract diluted with tap water in the following proportions: 25 mL extract + 975 mL tap water (V1), 50 mL extract + 950 mL tap water (V2) and 100 mL extract + 900 mL tap water (V3). Given that the density of the extract was approximately 1 g cm^−3^, it can be approximated that the dilution of the extract was 2.5% (V1), 5% (V2) and 10% (V3). The control was designated as V0.

A qualitative analysis (phycochemical screening) was conducted on the aqueous extract of *F. vesiculosus* (the base extract prior to dose preparation). The results of the tests are presented in Table 1. The following methods were used.

FeCl_3_ test [40,41,42]. To the diluted extract, 3–4 drops of 10% FeCl_3_ were added; a blue colour was expected for the presence of tannins/phenolic compounds.

Gelatin test [43]. The extract of the formulation was treated with 1% gelatin solution containing 10% sodium chloride. The formation of a precipitate indicates the presence of tannins and phenolic compounds.

Alkaline test [40,42,43,44]. A few drops of 5% NaOH solution were added to 1 mL of raw extract (which was expected to produce a deep yellow colour). The colour was expected to be lost in the presence of dilute HCl in the case of flavonoid presence.

Lead acetate test [42,45]. A small quantity of each extract was treated with a few drops of lead acetate solution. The formation of a yellow-coloured precipitate after a few minutes indicated the presence of flavonoids.

Shinod test [46]. A total of 2 mL of the extract and a small magnesium chunk were warmed. A few drops of concentrated HCl were added; a red colour was expected for flavonoid presence.

Mayer test [41,47]. A total of 2 mL of the extract was treated with Mayer’s reagent (potassium mercuric iodide solution) (a few drops were added). The formation of a cream-coloured precipitate was expected in the presence of alkaloids.

Wagner test [42,43,48,49]. A total of 2 mL of the extract was mixed with 1 mL of, and then treated with a few drops of Wagner’s reagent (a solution of iodine in potassium iodide). The formation of a reddish-brown precipitate was expected in the presence of alkaloids.

Dragendorff test [41,44,47,48,50]. To a 2 mL portion of the extract, Dragendorff’s reagent (potassium bismuth iodide solution) (a few drops) was added; an orange-red precipitate was expected in the presence of alkaloids.

Keller–Killiani test [43,48,50]. A total of 1 mL of acetic acid and 2 drops of ferric chloride were added to 2 mL of the extract, then 1 mL of concentrated sulphuric acid. A colour change to reddish-brown was expected in the presence of cardiac glycosides.

Liebermann–Burchard test [47,48]. To 2 mL of extract, Liebermann’s reagent was added—the reaction of triterpenoid with Liebermann’s reagent is expected to produce a red-purple colour, while steroid gives a green-blue colour.

Salkowski test [44,48,50]. H_2_SO_4_ and 2 mL of crude extract were shaken with 1 mL of CHCl_3_. A few drops of concentrated sulphuric acid were added to these solutions along the side of the test tube. The presence of a red-brown colour at the interface is expected in the presence of triterpenoids.

Fehling test [40]. To 2 mL of extract, a 2 mL mixture of Fehling’s solutions A and B (a ratio of 1:1) was added, which was heated in a water bath for a few minutes. The formation of a brick-red precipitate is expected in the presence of reducing sugars.

Biuret reaction [47]. To the extract, 1 mL of 40% sodium hydroxide solution and two drops of 1% copper sulphate solution were added. The formation of a violet colour is expected in the presence of proteins.

Xanthoproteic reaction [51]. To the extract, 1 mL of concentrated nitric acid was added. Then, 20% of sodium hydroxide or ammonia was added. An orange colour indicates the presence of aromatic amino acids.

Borntager test [50,52]. A total of 2 mL aqueous solution of ammonium hydroxide at 10% was added to 3 mL extract and mixed. Then, 10% *v*/*v* NH_4_OH solution was added. The appearance of pink, violet or red colouration is expected in the presence of anthraquinones.

Molish test [40]. A few drops of Molisch’s reagent (α-naphthol 1% (*w*/*v*) in 90% ethanol) were added to 20 mL of the extract, then 1 mL of concentrated H_2_SO_4._ The formation of a violet or red colour at the interphase of the two layers is expected in the presence of carbohydrates.

Foam test [40,41,43,44,47,48]. About 0.5 mL of the extract and 5 mL of distilled water were combined and agitated. Then, the formation of foam confirmed the presence of saponins. In a test tube, 10 mL deionised water was added to 5 mL extract. It was vigorously agitated by a vortex shaker for 2 min to form foam. The reaction is positive if foam persists in test tubes after resting for 15 min.

Emulsion test [47]. To 2 g of each plant extract, 2 mL of distilled water was added in test tubes. The solution was shaken vigorously and observed for a stable persistent foam. It was then mixed with 3–4 drops of sunflower oil and again shaken vigorously; after that, it was observed for the formation of emulsions.

### 4.3. Pot Experiment

The plants (*Hordeum vulgare* L., TORAF, Kluczbork, Poland) were sown and cultivated in a controlled pot experiment. The air temperature was maintained at 20 °C, with humidity levels set at 80%. The growth chamber emitted photosynthetically active radiation within the range of approximately 400 to 700 nm, with an intensity of 800 µmol m^−2^/s^−1^. The photoperiod was 10 h of light and 14 h of dark. The substrate was a loose quartz mineral mix with a pH of 6.0. It had 9.39 mg of P_2_O_5_ and 20.8 mg of K_2_O per 100 g. The seeds utilised in the experiment were intended for sprouting and had obtained the requisite organic farming certification. The seeds were produced in accordance with European Union standards, specifically without the use of chemicals or mineral fertilisers. The plants were irrigated with a fresh control solution, namely tap water (V0), as well as solutions of water containing labelled *F. vesiculosus* algae extracts, designated V1, V2 and V3. Each experimental object comprised 20 pots. The pot experiment employed a completely randomised design, with irrigation occurring every 24 h for a period of seven days. The experiment was conducted for eight days from the time of germination, with all experimental objects removed simultaneously. The microgreens (approximately 6 g from each pot) were then harvested and further assayed.

### 4.4. Assay of Microgreens

#### 4.4.1. Determination of Enzymatic Activity

The following section outlines the methodology employed in the determination of enzymatic activity.

A sample was taken for the analysis of superoxide dismutase (SOD), catalase (CAT), polyphenol oxidase (PPO) and phenylalanine ammonia. The determination of enzymatic activity was conducted using a sample prepared by homogenising 100 mg of frozen tissue (held at −70 °C) and grinding. The sample was analysed for superoxide dismutase (SOD), catalase (CAT), polyphenol oxidase (PPO) and phenylalanine ammonia-lyase (PAL) activity. The tissue was then homogenised in 1 mL of 0.9% NaCl containing 0.05% Triton X-100, 1% polyvinylpyrrolidone and a protease inhibitor cocktail, and centrifuged at 15,000× *g* for 30 min at 4 °C. The resulting supernatant was then subjected to analysis in order to ascertain the enzymatic activity. The activity of SOD was determined using a spectrophotometric method based on the inhibition of epinephrine autoxidation, as described by Piechowiak and Balawejder [53]. In brief, 5 µL of the enzyme extract was combined with 95 µL of a 50 mM sodium carbonate buffer (pH 10.2) and 10 µL of a 20 mM epinephrine solution in 0.1 M HCl. The kinetics of the absorbance increase were measured at a wavelength of 490 nm. One unit of SOD activity was defined as the amount of protein required to inhibit the epinephrine autooxidation by 50%. The activity of catalase (CAT) was determined using ammonium metavanadate in accordance with the methodology described by Hadwan and Ali [54], with a minor modification. In brief, 5 µL of the enzyme extract was added to 95 µL of phosphate buffer (pH 7.0) and 30 µL of 10 mM hydrogen peroxide. Following a five-minute incubation period at 37 °C, the reaction was terminated by the addition of 30 µL of a 10 mM ammonium metavanadate solution in 0.5 M sulphuric acid. The rate of increase in absorbance at 420 nm was determined. The term “unit of PPO” was defined as the quantity of enzyme that resulted in an increase in absorbance of 0.01 within one minute. The activities of the aforementioned enzymes were standardised to 1 mg protein, as determined by the Bradford method [55]. Twenty samples were obtained from each experimental variant. Each measurement was conducted in triplicate. Absorbance was measured at a wavelength of 452 nm. The unit of CAT activity was defined as the amount of enzyme required to cause a decrease in absorbance of 0.01 units within a one-minute incubation period. To determine the activity of phenylalanine ammonia-lyase, a solution of 100 µL of 50 mM borate buffer (pH 8.0) was prepared and combined with 50 µL of 50 mM L-phenylalanine and 50 µL of the enzyme extract. The enzymatic mixture was incubated at 37 °C for one hour, after which the reaction was terminated by the addition of 10 µL of 6 M hydrochloric acid. One unit of PAL activity was defined as the amount of enzyme required to increase the absorbance at 290 nm by 0.01 within one hour. The activity of PPO was determined through a colourimetric assay utilising pyrocatechol as the substrate. To this end, 100 µL of 50 mM sodium phosphate buffer (pH 6.4), 20 µL of the enzyme extract and 80 µL of 1% pyrocatechol were added. The kinetics of the increase in absorbance at 420 nm were determined. 

#### 4.4.2. Analysis of Antioxidant Status and Phenolic Compounds

The initial step in the analysis of antioxidant activity entailed the freeze-drying of the samples, which were initially frozen at −40 °C and subsequently subjected to freeze-drying at a temperature of 30 °C and a vacuum of 500 mTorr. Subsequently, the samples were milled for 15 s using a laboratory grinder. They were then homogenised with 1 mL of a 75% methanol solution (50 mg of lyophilisate) and centrifuged at 10,000× *g* for 30 min at 4 °C. Subsequently, the supernatant was employed for the analysis of antioxidant status. The antioxidant activity was quantified in terms of Trolox equivalents, with the results expressed as milligrams of Trolox equivalents per gram of dry matter. In brief, 2 µL of the supernatant was combined with 198 µL of a DPPH methanol solution (diluted to an A515 value of 1.0 ± 0.3) and incubated in the dark for 30 min at room temperature. The absorbance was determined using a microplate reader at a wavelength of 515 nm. The calibration curve was found to have the following form: y = −1.4068x + 1.315, R^2^ = 0.9994. For the determination of total phenolic content, 10 µL of the supernatant was pipetted into the well of a plate containing 90 µL of distilled water, 20 µL of Follin–Ciocalteu reagent (diluted 1:1 with distilled water) and 30 µL of 20% sodium carbonate. Following a 30 min incubation period in the absence of light, the solution’s absorbance was determined at a wavelength of 700 nm. The calibration curve was found to have the following form: y = 4.0896x + 0.039, R^2^ = 0.9992. The results were expressed as gallic acid equivalents per gram of dry matter (mg GAE g^−1^ d.m.). The flavonoid content was analysed using a colourimetric method based on the formation of complexes between flavonoids and aluminium chloride in an alkaline medium. In summary, 20 µL of the extract was combined with 80 µL of methanol, 10 µL of 5% sodium nitrite, 10 µL of 10% aluminium chloride, 100 µL of sodium hydroxide and 30 µL of distilled water. Following a 15 min incubation period, the absorbance was monitored at 430 nm. The calibration curve was found to have the following form: y = 2.1261x + 0.0748, R^2^ = 0.9987. The results were expressed as quercetin equivalents [mg QE g^−1^]. All obtained results were converted to 1 g of dry matter (d.m.) of the sample, which was determined by drying the sample at 105 °C using a moisture analyser (RADWAG, Radom, Poland). Twenty samples were taken from each variant of the experiment, and each measurement was repeated three times.

#### 4.4.3. Statistical Analysis

One-way analysis of variance (ANOVA) and Tukey’s post hoc test were performed using STATISTICA 13.1 software (TIBCO Software Inc., Hillview Avenue, Palo Alto, CA, USA) at a significance level of α = 0.05. The statistical means of each experimental object (with standard deviation SD) were compared between experimental variants to analyse the results.

## 5. Conclusions

The increasing global demand for food and the fear of the excessive use of synthetic chemicals in agriculture have led the sector to seek sustainable solutions to maintain or improve crop yields under stress conditions caused by global change. Brown algae are being extensively researched for their ability to produce a variety of bioactive compounds, including those that regulate osmosis, act as antioxidants, promote bacterial growth and stimulate plant growth. Brown algae such as *Fucus vesiculosus* (bladderwrack) contain phenolic compounds, fucoxanthin, minerals and bioactive polysaccharides. These compounds have significant biological properties and can act as plant biostimulants, promoting growth and stress tolerance.

The objective of the study was to assess the content of phenolic compounds (TPC) in barley microgreens following irrigation with *F. vesiculosus* extract. The findings indicated that the extract had no notable impact on TPC. The highest TPC was observed at a concentration of 5%, although the differences were not statistically significant. The flavonoid content remained unaltered, which may be attributed to the absence of suitable chemical compounds in the extract or the particular characteristics of the plant. The antioxidant activity demonstrated an increase following irrigation with the extract, reaching a maximum at a concentration of 5%. However, a slight decrease was observed at 10%. The reduction in antioxidant activity at the V3 dose in comparison to V2 may be attributed to a saturation effect, unfavourable chemical interactions or toxic effects associated with higher doses of the extract. The highest PPO activity was observed at a concentration of 5%, while the highest PAL activity was observed at a dose of 10%. The application of the extract at concentrations of 2.5 and 5% resulted in a reduction in PAL activity. The application of *F. vesiculosus* extract at concentrations of 5% and 10% resulted in an increase in the concentration of SOD in barley microgreens. The highest catalase activity was observed in the control, while the lowest was observed in the 5% extract irrigation. Doses of 2.5% and 5% caused a decrease in catalase activity compared to the control, while the 10% dose did not significantly change catalase activity. The highest SOD levels were observed at the 5% and 10% doses, suggesting that these doses are most effective in increasing SOD levels and protecting plants from oxidative stress.

Overall, brown algae are highlighted as a valuable natural resource with multiple applications that warrant further research and sustainable use.

## Figures and Tables

**Table 1:** Qualitative analysis (phytochemical screening) of the extract.

Test Name	Phytochemical Group Detected	Sample Name	Interpretation	Result Obtained
FeCl_3_ test	tannins	10B	dark brown gelatinous precipitate	-
gelatin test	tannins/phenolic compounds	10C	no chemical reaction	-
alkaline test (NaOH)	anthocyanins/flavonoids	10D	no chemical reaction	-
lead acetate test	tannins/flavonoids	10E	white gelatinous precipitate	inconclusive
Shinod test	flavonoids	10F	solution brightened, foam appeared	-
Mayer test	alkaloids	10G	precipitate after adding HCl	-
Wagner test	alkaloids	10H	no chemical reaction	-
Dragendorff test	alkaloids	10I	precipitate after adding HCl	-
Keller–Killiani test	cardiac glycosides	10J	very subtle darkening at the junction of two phases	inconclusive
Liebermann–Burchard test	cardiac glycosides/steroids/terpenoids	10K	greenish ring	glycosides/steroidal aglycones +
Salkowski test	steroids/terpenoids	10L	very subtle darkening at the junction of two phases	inconclusive
Fehling test	reducing sugars/carbohydrates	10M	blue-green solution	-
Biuret reaction	proteins and amino acids	10N	gelatinous precipitate/green tint	-
Xanthoproteic reaction	proteins and amino acids	10O	after HNO_3_—no precipitate; after adding NH_3_—solution turns yellowish	-
Borntrager test	anthraquinones	10P	subtle pinkish tint	inconclusive
Molish test	reducing sugars/carbohydrates	10R	no chemical reaction	-
foam/emulsion test	saponins	10A	foaming, emulsifying and clouding were found	+

## Data Availability

The data presented in this study are available in this article.
